# Chemical Profiles and Antimicrobial Activities of Essential Oil From Different Plant Parts of Fennel ( *Foeniculum vulgare* Mill.)

**DOI:** 10.1002/fsn3.70307

**Published:** 2025-05-19

**Authors:** Omer Elkiran, Ozge Telhuner

**Affiliations:** ^1^ Department of Medical Services and Techniques Vocational School of Health Services, Sinop University Sinop Türkiye

**Keywords:** antimicrobial activity, essential oil, estragole, fenchyl acetate, *Foeniculum vulgare*

## Abstract

The aim of this study was to investigate the chemical composition and antimicrobial activities of essential oils obtained from different organs (leaf, stem, flower, and seed) of *Foeniculum vulgare* (fennel) plant naturally growing in Sinop province of Turkey. Plant samples collected from the field in 2023 were dried and their essential oils were isolated using the hydro‐distillation method with a Clevenger type device. Chemical analyzes were performed simultaneously with GC‐FID and GC–MS devices, and component identifications were made with the help of NIST and Wiley libraries. According to the results, estragole (methyl chavicol) was determined as the main component of leaf (51.7%), flower (38.9%) and seed (53.3%) essential oils. Fenchyl acetate (35.3%) was the dominant component in the stem essential oil. In addition, other important compounds such as limonene, fenchon, and γ‐terpinene were detected in different proportions. It is thought that these composition differences between organs are due to environmental factors and plant physiology. Within the scope of biological activity studies, the antimicrobial effects of essential oils obtained from each organ against seven Gram‐positive and Gram‐negative bacteria and two fungi were tested by disk diffusion and minimum inhibitory concentration (MIC) methods. It was observed that Gram‐positive bacteria were particularly more sensitive. The highest antibacterial effect was obtained against *Staphylococcus aureus* with a 30 mm inhibition zone in the seed oil. According to MIC values, the lowest effective concentration was seen on *B. cereus* , *B. subtilis* , and *S. aureus* with 50 μg/mL. No significant effect was observed against *A. niger* and *E. faecalis* . This study is the first to reveal the chemical and biological properties of fennel essential oils obtained from different organs in Türkiye.

## Graphical abstract

This study examines essential oils' chemical profiles and antimicrobial activities derived from different parts of Fennel ( *Foeniculum vulgare* Mill.). The results highlight the oils' effectiveness against various microorganisms and their potential therapeutic applications.

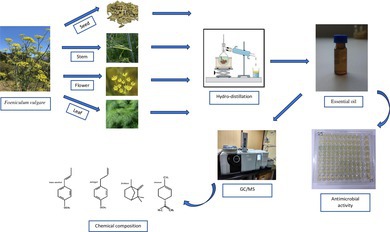

## 1 Introduction

Apiaceae is one of the largest plant families with 450 genera and 3700 species (Amiri and Joharchi 2016). Fennel ( *F. vulgare* ) is a perennial, medicinal, and aromatic plant in the Apiaceae family (Mokhtari and Ghoreishi 2019). Although fennel grows naturally in Southern, Western Anatolia, and Northern Anatolia, it is also cultivated in the West and South (Caliskan et al. 2014). Fennel is considered one of the oldest medicinal herbs and has been used for over 4000 years. Since ancient times, laxatives have been used for many purposes such as treating menstrual disorders, indigestion problems, bloating, and cough, as well as reducing the painful effects. While the fruits, seeds, and young leaves of the fennel plant are used to flavor meals, its brewed fruits are also used as a carminative, and its roots are used as a laxative. Boiled seeds are used to regulate menstruation and diuretics. Fennel plant poultice is used to relieve breast swelling in breastfeeding mothers. Its seeds are also used as an antipyretic, cough reliever, treatment of venereal diseases, and to facilitate birth. The oil of the plant is used for bloating and intestinal worms (Al‐Snafi 2018).

Plants produce primary metabolites such as nucleic acids, proteins, carbohydrates, and fats that have a direct role in growth and development, and secondary metabolites that do not have a direct role in vital activity (Tiring et al. 2021). Although secondary metabolites do not have vital activities in the growth and development of plants, the therapeutic properties found in medicinal and aromatic plants originate from these metabolites. Studies have found that there are more than 30,000 secondary metabolites in plants (Bati 2011). Secondary metabolites are small molecule metabolites such as alkaloids, essential oils, glycosides, phenols, steroids, and colorants (Baydar 2013).

Essential oils are fragrant, volatile, and oily liquids that occur naturally in medicinal and aromatic plants, are biologically active, and are produced by secondary metabolism (Cadena et al. 2018). The activities of essential oils and the effects of plants should not be confused with each other. Essential oils have antiseptic, antioxidant, antifungal, digestive stimulant, antitoxigenic, insecticidal, antiviral, antiparasitic, anti‐inflammatory, and antibacterial properties (Bayaz 2014). The source of the antimicrobial effect of essential oils is that they contain many different components in their structures (Chouhan et al. 2017). Although the mode of action and antimicrobial activities of essential oils are related to the chemical structure of the components in the essential oil, the effects of antimicrobial agents vary depending on the types of microorganisms. This difference is related to the structure of the cell wall of microorganisms and the structure of the outer membrane (Sayin 2019).

The most important chemical components of the essential oils of the fennel plant are trans‐anethole, estragole (methyl chavicol), fenchone, and α‐phellandrene and have many applications in the food, pharmaceutical, and health sectors. These essential oils have anti‐hypertensive, anti‐spasmodic, anti‐inflammatory, blood pressure lowering, and analgesic properties (Mokhtari and Ghoreishi 2019).

The aim of this study is to determine the chemical compositions of the essential oils of different organs (leaf, stem, seed and flower) of the fennel plant ( *F. vulgare* ), which grows naturally in Sinop province, and to determine the biological activities of essential oils and their level of influence from environmental factors.

## 2 Material and Methods

### 2.1 Materials

#### 2.1.1 Plant Material

The plant samples were collected from Sinop in 2023, taking into account population densities. Plant samples were described by Dr. O. Elkiran using the Flora of Turkey and East Aegean Islands (Volume 4) (Davis 1972). The collected plant samples were kept in a shaded place for an average of 1 week before the study began.

#### 2.1.2 Isolation of Essential Oil

The aerial parts of the plant (stem, leaf, flower and seed) were obtained by the hydrodistillation method using the Clevenger device in 3 h. The obtained essential oils were placed in colored vials and kept at +4°.

#### 2.1.3 Microorganisms

Gram‐positive bacteria *B. cereus* ATCC 14579, *B. subtilis* ATCC 6623, *S. aureus* ATCC 25923, *E. faecalis* ATCC 29212; the Gram‐negative bacteria *E. coli* ATCC 25922, *K. pneumoniae* ATCC 70060, *P. aeruginosa* ATCC 27853; the fungi *C. albicans* ATCC 1023 and *A. niger* ATCC 16404 were used in the antimicrobial activity study. All test types were obtained from the ATCC (American Type Culture Collection) culture collection.

### 2.2 Methods

#### 2.2.1 Chromatographic Analysis

Essential oil analysis was performed using Shimadzu GCMS QP 2010 ULTRA brand/model device. The percentage of chemical components in the essential oil was calculated by taking into account the GC/FID peaks.

In the study, RXI‐5MS capillary column (30 m × 0.25 mmi. d., film thickness 0.25 μm) was used as helium carrier gas, the flow rate was 1 mL/min and the injector temperature was 250°C. The ambient temperature in GC was 40°C for the first 4 min and 240°C for the next 53 min. Electronic libraries (Wiley and NIST) were used in the identification of the components in the essential oil (McLafferty and Stauffer 1989; NIST 2023).

#### 2.2.2 Evaluation of Antimicrobial Activity

The antibacterial and antifungal activity of essential oil samples taken from four different parts of the fennel plant (stem, seed, flower and leaf) was evaluated using the disk diffusion method (Bauer 1966; Elkiran, Avsar, Veyisoglu, et al. 2023). All microorganisms were kept at −80°C until studied in stock tubes containing 25% (*v*/*v*) glycerol. Muller Hinton Agar (MHA) was used to activate bacterial cultures, and Sabouraud Dextrose Agar (SDA) (Difco) was used for fungi. Before starting the study, bacteria were allowed to grow overnight in Mueller Hinton Broth at 37°C, and fungi were allowed to grow overnight in Sabouraud Dextrose Broth at 28°C. The turbidity of the prepared suspensions of the test strains was adjusted to 0.5 McFarland equivalent (1.5 × 10^8^ cfu/mL). Then, the microorganism was spread on the surface of the agar plate with 100 μL sterile swabs. Filter paper disks (6 mm) were loaded with 25 μL of essential oil samples and kept in laminar flow to dry completely. After drying, the petri dishes containing bacteria were incubated at 37°C overnight, and the petri dishes with fungus cultivation were incubated at 28°C. Pure water was used for negative control, Ampicillin (AM10), Gentamicin (CN10) for positive control, and Cycloheximide (Cyc) for fungi.

Minimum Inhibitory Concentration (MIC) was determined using the broth microdilution method in 96‐well microplates as described in the National Committee for Clinical Laboratory Standards (Wayne 2007). Stock solutions of essential oils (800–3125 μL/mL) were diluted into MHB (for bacteria) and SDB (for fungi) in glass tubes at different concentrations. In the study, pure microorganisms and pure media placed in the wells were determined as the control group. Each microorganism and compound were placed in the wells in 100 μL amounts. It was recorded that no microorganism growth was observed on the microplate, representing the MIC expressed in μg/mL. The experiment was performed in duplicate.

## 3 Result and Discussion

### 3.1 Chemical Composition of Essential Oil of *F. vulgare*

**FIGURE 1:**
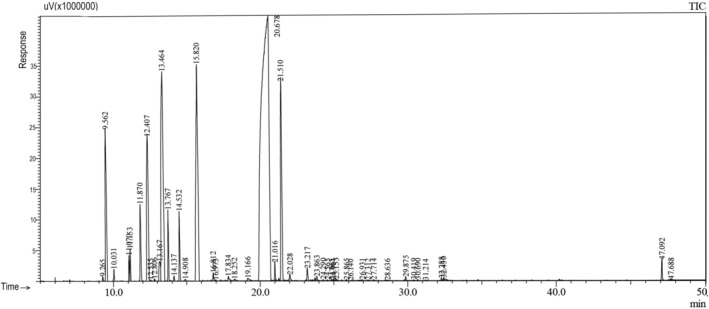
Gas chromatography‐flame ionization detector (GC‐FID) profile of the essential oil of the leaf of *Foeniculum vulgare*.

**FIGURE 2:**
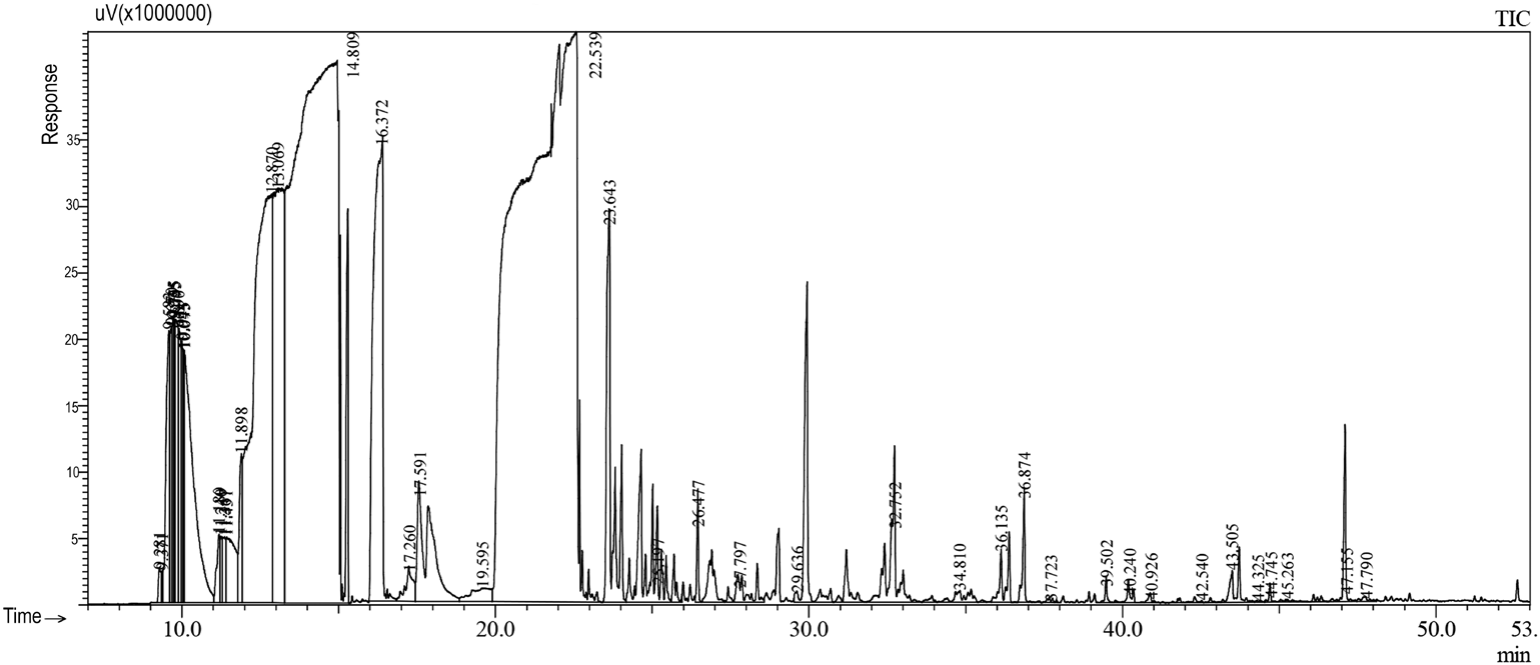
Gas chromatography‐flame ionization detector (GC‐FID) profile of the essential oil of the stem of *Foeniculum vulgare*.

**FIGURE 3:**
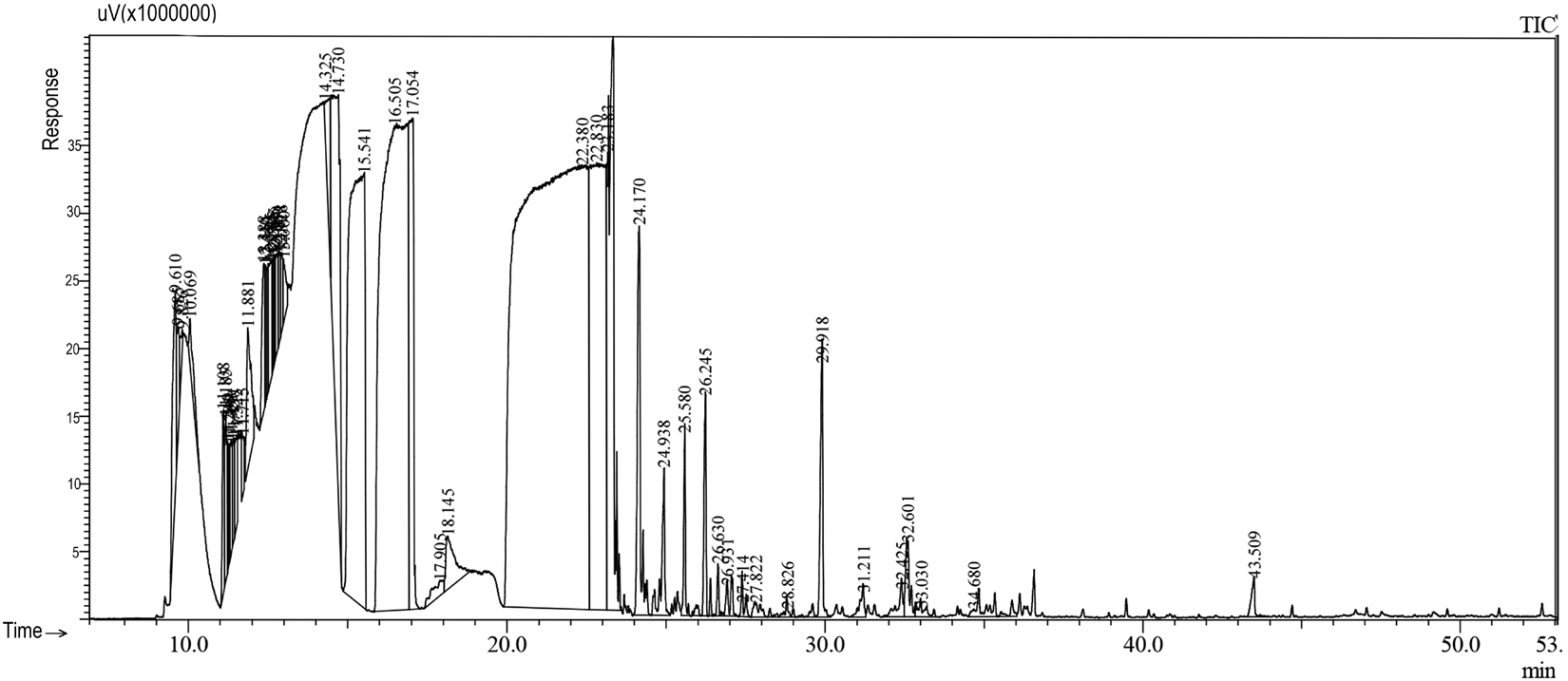
Gas chromatography‐flame ionization detector (GC‐FID) profile of the essential oil of the flower of *Foeniculum vulgare*.

**FIGURE 4:**
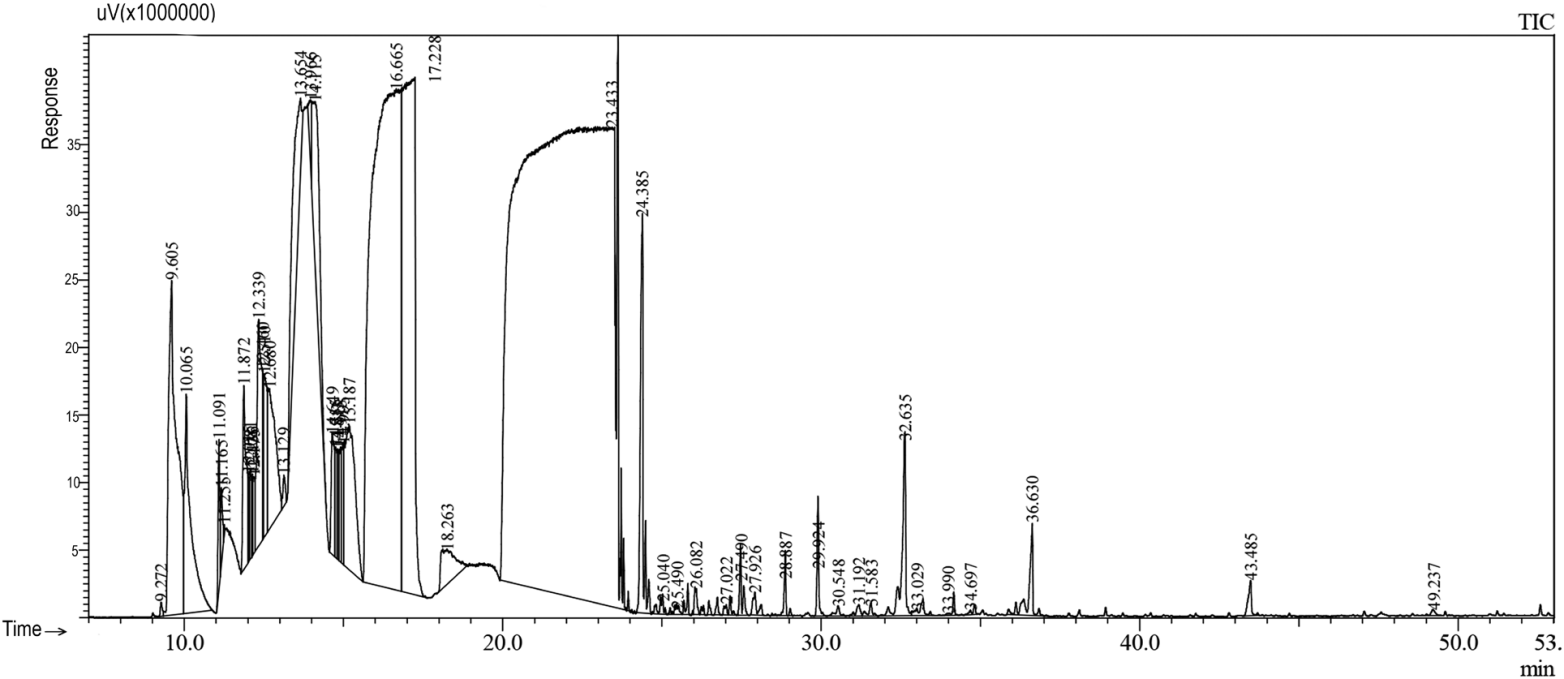
Gas chromatography‐flame ionization detector (GC‐FID) profile of the essential oil of seed of *Foeniculum vulgare*.

In the study, an average of 1–1.5 mL of essential oil was extracted from different organs of fennel (leaf, stem, flower, and seed). It has been observed that the chemical components and yields of the essential oils obtained vary in each organ. According to our study results, it was determined that the main component in leaf, flower, and seed essential oils was estragole (methyl chavicol) (51.7%; 38.9%; 53.3%, respectively), while the main component in the stem was fenchyl acetate (35.3%) (Table 1, Figures 1, 2, 3, 4). It has been observed that the main component is the same in leaf, flower, and seed essential oils, but the main component changes in the stem. According to literature data, fennel essential oil study results are similar to our results, but the main component is generally trans‐anethole (Stefanini et al. 2006; Kan 2006; Sanli et al. 2008, 2012; Singh et al. 2006; Miguel et al. 2010; Karayel 2019; Acikgöz and Kara 2020). In addition, while there are many studies on the aboveground organs of fennel (as a whole), essential oil studies on an organ basis, as we did, are limited. Our organ‐based results are discussed along with the results of existing studies.

**TABLE 1:** Essential oil composition of aerial parts of *Foeniculum vulgare* .

No	RRI	References	Compounds	Leaves RA (%)	Stem RA (%)	Flowers RA(%)	Seeds RA(%)
1	1027	997–1027^a^	3‐Carene	—	1.0	1.0	—
2	1039	998–1029^a^	Tricyclene	—	0.5	0.5	—
3	1063	1008–1039^a^	α‐Pinene	—	—	—	3.30
4	1064	1052–1074^a^	cis‐Sabinene hydrate	—	0.20	—	—
5	1066	1014–1188^b^	*α*‐Terpinene	4.40	—	—	—
6	1070	953–1076^c^	Camphene	—	—	0.20	1.70
7	1071	1063^d^	*a*‐Fenchene	—	3.30	—	—
8	1095	1118^e^	*β*‐Pinene	0.4	—	0.7	—
9	1099	1132^f^	Sabinene	0.4	0.9	0.8	0.2
10	1110	1174^b^	Myrcene	1.70	0.40	1.00	0.70
11	1122	1176^e^	*α‐*Phellandrene	6.10	—	0.50	1.80
**12**	**1132**	**1126–1149** ^a^	***trans*‐Limonene oxide**	—	**8** **.50**	—	—
13	1136	1250^b^	Geraniol	—	4.60	—	—
14	1139	1001–1076a	o‐Cymene				
**15**	**1149**	**1163** ^g^	***trans‐β‐*terpineol**	—	—	—	**5.10**
**16**	**1175**	**1097** ^h^	**Limonene**	**11.50**	**26.80**	**4.20**	**1.50**
**17**	**1191**	**1057–1256** ^c^	**ɣ‐Terpinene**	**1.20**	—	**9.00**	**1.70**
**18**	**1197**	**1084–1290** ^b^	**Terpinolene**	**10.50**	**0.90**	—	—
**19**	**1209**	**1219–1220** ^i^	***endo*‐Fenchyl acetate**	—	**4.60**	—	—
**20**	**1228**	**1078–1378** ^j^	**L‐Fenchone**	—	—	**19.40**	**24.50**
21	1229	1236^k^	trans‐Carveol	—	0.2	—	—
22	1236	1132^m^	allo‐Ocimene	1.20	1.60	—	—
23	1251	1145–1532^c^	Camphor	—	—	0.6	0.6
24	1280	1170–1211^a^	Naphthalene	—	0.3	—	—
**25**	**1304**	**1191–1687** ^n^	**Estragole (Methyl chavicol)**	**51.7**	—	**38.9**	**53.3**
**26**	**1322**	**1233** ^o^	**Fenchyl acetate**	**6.60**	**35.30**	**4.60**	—
**27**	**1351**	**1329–1358** ^a^	***α*‐Terpinyl acetate**	—	—	**8.80**	—
28	1369	1264–1297^a^	Bornyl acetate	—	2.00	—	—
29	1381	1280^p^	Anethole	—	—	1.60	1.30
30	1397	1350^q^	Anisketone	—	—	0.6	—
31	1403	1419^k^	α‐Humulene	—	1.40	—	—
32	1426	1376–1401^a^	Dodecanal	—	—	0.8	—
33	1431	1363–1391^a^	*α‐*Copaene	—	0.5	—	—
34	1460	1413–1463^a^	Aromadendrene	—	0.3	—	—
35	1501	Not found	9‐Decen‐2‐one, 5‐methylene—	—	2.30	—	—
36	1507	1464–1493^a^	Germacrene D	—	—	0.9	—
37	1566	1565^k^	Dodecanoic acid	—	—	0.4	0.7
38	1612	1563–1595^a^	Caryophyllene oxide	—	0.20	0.30	0.40
39	1660	1669–1707^a^	*β‐*Sinensal	—	0.3	—	—
40	1805	1784–1814^a^	Hexadecanal	—	0.2	—	—
41	1884	1778–1854^a^	Germacrene B	0.4	0.4	—	—
Grouped compounds (%)							
Monoterpene hydrocarbons				37.4	55.5	46.7	41.1
Oxygenated monoterpenes				58.3	35.6	45.9	54.6
Sesquiterpenes hydrocarbons				0.4	3.3	1.6	1.1
Others				—	2.3	0.6	—
**Total identified compounds (%)**				**96.1**	**96.7**	**94.8**	**96.8**

Abbreviations: RA, relative area (peak area relative to the total peak area); Ref., references; RRI, relative retention indices.

^a^ Babushok et al. (2011).

^b^ Bobakulov et al. (2020).

^c^ Jemia et al. (2013).

^d^ Tucker et al. (2000).

^e^ Tabanca et al. (2011).

^f^ Turkmenoglu et al. (2015).

^g^ Hend et al. (2018).

^h^ Dogan and Bagci (2018).

^i^ Ali et al. (2017).

^j^ Touati et al. (2011).

^k^ Elkiran, Avsar, and Bagci (2023).

^m^ Tognolini et al. (2007).

^n^ Verma et al. (2012).

^o^ Diao et al. (2014).

^p^ Raal et al. (2012).

^q^ Salami et al. (2016).

In fennel leaf essential oil, Estragole (methyl chavicol) (51.7%), limonene (11.5%), terpinolene (10.5%), and fenchyl acetate (6.6%) were determined as the main components, and a total of 13 components were detected (≥ 0, 25). Stefanini et al. (2006) and Katar et al. (2021) reported that limonene was the main component in the essential oil in the leaf (42.3%, 41.3%). Sanli et al. (2008) found that the essential oil components obtained from different parts of fennel differed throughout their development periods; the main component of the essential oil in fennel leaves was trans‐anethole (29.6%–38.5%), and other important components were α‐pinene (18.6%–19.1%), α‐phellandrene (17.6%–22.5%) and limonene (6.2%–8.3%). In our study, the presence of limonene (11.5%) among the main components of leaf essential oil is similar to the literature data.

The main components in the chemical content of fennel stem essential oil were determined as fenchyl acetate (35.3%), limonene (26.8%), trans‐Limonene oxide (8.5%), and endo‐Fenchyl acetate (4.6%), and a total of 24 components were identified (≥ 0.25). Acikgöz and Kara (2020) reported that trans‐anethole (41.8%–48.5%), fenchone (6.0%–7.5%), α‐pinene (11.2%–14.5%), and α‐phellandrene (7.0%–10.7%) were the main compounds of essential oil in different development periods of fennel. Also, Katar et al. (2021) reported estragole (methyl chavicol) (40.1%) as the main compound of the essential oil of Fennel stem. In another study, Stefanini et al. (2006) reported that limonene (42.3%) was the first major compound in the essential oil of the stem and leaf of *F. vulgare* . In the results of our study, the main compound was limonene (26.8%), as Stefanini et al. (2006) reported in their study.

The main components of the essential oil obtained from the flower of fennel are estragole (methyl chavicol) (38.9%), L‐fenchone (19.4%), Ɣ‐terpinene (9.0%), and α‐Terpinyl acetate (8.8%) and a total of 20 components were detected (≥ 0.25).

Acikgöz and Kara (2020) reported that the main components of the essential oil obtained from fennel flowers were trans‐anethole (54.7%), α‐pinene (11.7%), limonene (10.2%) and fenchone (7%). In another study, Katar et al. (2021) reported that the most important component of the essential oil of fennel flower was estragole (methyl chavicol) (71.0%). The main components in our study results were estragole (methyl chavicol) (38.9%) and L‐fenchone (16.6%), which are similar to these studies.

The main components of the essential oil obtained from fennel seeds were estragole (methyl chavicol) (53.3%), L‐fenchone (24.5%), trans‐β‐terpineol (5.1%), and α‐pinene (3.3%) were determined, and a total of 14 components were identified (≥ 0.25). Stefanini et al. (2006) determined that the main component was trans‐anethole (78.2%) of the seed essential oil content of fennel collected in the summer months. Sanli et al. (2008) reported that trans‐anethole (45.4%–76.0%), fenchone (4.6%–30.7%), and γ‐terpinene (1.1%–10.2%) were the main compounds in their study conducted in different periods. Marotti and Piccaglia (1992) reported that the main components of the essential oil obtained from fennel seeds were trans‐anethole (81.8%–91.1%), fenchone (1.3%–10.2%), and estragole (methyl chavicol) (1%, 5%–3.9%). Also, Anwar et al. (2009), in their study on seed essential oil, reported the main components as trans‐anethole (69.8%), fenchone (10.2%) and estragole (methyl chavicol) (5.4%). In addition, Diao et al. (2014), in their study on fennel seeds, stated that the main components of the essential oil are trans‐anethole (68.5%), estragole (methyl chavicol) (10.4%) and limonene (6.2%). Balkan (2015) reported the main components in seed essential oils as trans‐anethole (88.1%–89.5%) and estragole (methyl chavicol) (3.9%–4.6%), while Ben et al. (2021) reported the main components of the seed essential oil as trans‐anethole (81.2%), estragole (methyl chavicol) (76.2%) and fenchone (24.4%). The main components of the essential oil we obtained in our study, estragole (methyl chavicol) (53.3%) and L‐fenchone (24.5%), are similar to the literature findings.

### 3.2 Biological Activity of Essential Oils of *F. vulgare*

The antimicrobial activities of essential oil samples taken from four different parts of the fennel plant (stem, seed, flower, and leaf) against seven bacteria and two fungi were determined using the disk diffusion method and MIC procedure. The antimicrobial activities of essential oils measured by disk diffusion and dilution methods are shown in Tables 2 and 3.

**TABLE 2:** Inhibition zones (mm) of essential oil extracts taken from four different parts of *Foeniculum vulgare* (stem, seed, flower and leaf) using the disk diffusion method against the tested pathogenic microorganisms.

EO extract	*B. cereus*	*B. subtilis*	*S. aureus*	*E. faecalis*	*E. coli*	*K. pneumoniae*	*P*. *aeruginosa*	*A. niger*	*C*. *albicans*
Fennel stem	10	10	10	—	8	14	10	—	—
Fennel seed	20	20	30	7	15	10	10	—	14
Fennel flower	20	20	20	—	12	12	10	—	8
Fennel leaf	20	15	24	—	10	12	10	—	10
Ampicillin	35	35	28	10	15	40	—	*	*
Gentamicin	20	20	40	4	20	26	15	*	*

Abbreviations: *, not tested; —, not effect.

**TABLE 3:** Minimum inhibitory concentration (MIC, μg ml^−1^) of essential oil of *Foeniculum vulgare* from four different parts of the fennel plant (stem, seed, flower and leaf) against some pathogenic microorganisms.

EO extract	*B. cereus*	*B. subtilis*	*S. aureus*	*E. faecalis*	*E. coli*	*K. pneumoniae*	*P*. *aeruginosa*	*A. niger*	*C*. *albicans*
Fennel stem	100	100	100	—	100	100	100	—	—
Fennel seed	50	50	50	—	100	100	100	—	200
Fennel flower	50	50	50	—	100	100	100	—	400
Fennel leaf	50	100	50	—	100	100	100	—	200

Abbreviation: —, not effect.

When the disk diffusion results of essential oils obtained from four different parts of the fennel plant (root, seed, flower, and leaf) were examined, it was determined that among the tested microorganisms, gram‐positive bacteria were more sensitive to essential oils than gram‐negative bacteria and fungi. Inhibition zones revealed the different sensitivity of different microorganisms to essential oils obtained from different regions (Table 2). While seed essential oil showed the best antibacterial effect against *S. aureus* from gram‐positive bacteria with a zone diameter of 30 mm, roots, flowers, and leaves showed zone diameters of 10, 20, and 24 mm, respectively. Seed essential oil showed the best antibacterial effect against *E. coli* (15 mm) from gram‐negative bacteria. Like the result we obtained in our study, many previous studies have reported lower sensitivity of Gram‐negative bacteria to essential oils (Dorman and Deans 2000; Omulokoli et al. 1997; Paster et al. 1990; Roby et al. 2013; Ilić et al. 2019). Additionally, essential oils showed high antimicrobial activity against some pathogens, equal to others, and lower to others compared to some standard antibiotics used in the study (Table 2).

Additionally, disc diffusion and MIC results were consistent in the study. Minimal Inhibitory Concentration (MIC) tests for essential oil samples were studied as 800–3.125 μL/mL (Table 3). The low MIC values of seed, flower, and leaf essential oils to *B. cereus* , *B. subtilis*, and *S. aureus* (50 μg/mL) indicate the highest susceptibility of these microorganisms. No essential oil showed any effect against *E. faecalis* and *A. niger* , among the other studied microorganisms.

The results of the biological activity study conducted with the seeds of the fennel plant grown in Pakistan are similar to our study results (Anwar et al. 2009). Our study results showed that the essential oils we obtained from different organs of the fennel plant were effective in all groups we tested, with the best effect on Gram‐positive bacteria. In another study, Ozcan et al. (2006) found that fennel essential oils showed an inhibitory effect against many *Bacillus* species. In a different study, when the antibacterial activity, minimum inhibitory concentration, and minimum bactericidal concentration of fennel seed essential oils were examined, it was seen that bacteria had different sensitivities (Diao et al. 2014).

## 4 Conclusion

The EO isolated from different organs of *F. vulgare* was found to be rich in Estragole and fenchyl acetate. The chemical results of this study might be helpful in terms of chemotaxonomy, potential usefulness, and cultivation of *Foeniculum* taxa. Microbiological tests of the studied sample may be helpful after further testing in practice. Future studies are needed to verify which constituents of the EO have antimicrobial or antioxidant activity.

## Author Contributions

**Omer Elkiran:** conceptualization (equal), data curation (equal), formal analysis (equal), investigation (equal), methodology (equal), project administration (lead), resources (equal), software (equal), supervision (lead), validation (equal), visualization (equal), writing – original draft (equal), writing – review and editing (lead). **Ozge Telhuner:** conceptualization (equal), data curation (equal), formal analysis (equal), investigation (equal), methodology (equal), resources (equal), software (equal), validation (equal), visualization (equal), writing – original draft (equal).

## Conflicts of Interest

The authors declare no conflicts of interest.

## Data Availability

The data that support the findings of this study are available on request from the corresponding author. The data are not publicly available due to privacy or ethical restrictions.
